# The relationship between mental health and violence toward women during the COVID-19 pandemic

**DOI:** 10.1186/s12888-022-04448-3

**Published:** 2022-12-12

**Authors:** Najmeh Khatoon Shoaei, Neda Asadi, Mahin Salmani

**Affiliations:** 1 Nursing Research Center, Kerman University of Medical Sciences, Kerman, Iran; 2 Department of Mathematics and Statistics, Faculty of Science, University of New Brunswick, Fredericton, Canada

**Keywords:** Mental Health, Violence toward women, The COVID-19 pandemic

## Abstract

The COVID-19 pandemic has a number of psychological consequences for societies, especially women. This study was conducted to determine the relationship between mental health and violence toward women during the COVID-19 pandemic in Iran.This study was conducted on during late October to November 2020 (*N* = 400). Demographic information questionnaire, General Health Questionnaire(GHQ-12) and violence toward women inventory(VTWI) were used.The results showed that violence was higher among employed women. Also, the results showed that VTW was higher in women with 3 children, high school degree, family income under 3 million and women over 40 years old. Findings showed that the mean mental health of women at the COVID-19 pandemic was moderate (15.14 ± 8.8). Also, with increasing psychological and economic violence, their mental health decreases. Therefore, it is suggested that policy makers and planners, apart from the physical effects of the COVID-19 pandemic, pay attention to its psychological dimension, especially for women, and try to allocate funds to maintain and promote mental health and family.

## Introduction

The COVID-19 pandemic originated in Wuhan, China in December 2019 and spread rapidly, becoming a global pandemic [1]. The spread of the disease worldwide was so rapid that it became the biggest public health threat in 2020. The COVID-19 pandemic has caused fear, insecurity and public anxiety in many parts of the world [2] and it has imposed a heavy burden on societies in terms of economic, social and mental health [3].

According to the World Health Organization (WHO), mental health is “a state of well-being in which the individual realizes his or her own abilities, can cope with the normal stresses of life, can work productively and fruitfully, and is able to make a contribution to his or her community” [4]. Research has shown that the spread of the COVID-19 pandemic has caused mental health problems for people. The study by Qiu et al. showed that during outbreak of the COVID-19 pandemic, more than half of people experienced moderate to high psychological disorders [5]. According to the study by Li Wen et al. (2020), people, especially women who have experienced long lockdown in the COVID-19 pandemic, faced family conflicts, behavioral and psychological problems in the family environment [6].

One of the potential family impacts of the COVID-19 pandemic that quickly attracted global attention is the spread of violence toward women. In this regard, the United Nations issued a statement on 27march 2020 and warned against its increase [7]. Violence toward women is includes all offensive and repressive behaviors such as, physical and/or sexual violence and psychological attacks, verbal and derogatory attacks, obstruction of communication with family and friends [8].WHO reported that65-92% of women are abused by their partners and 60% of women in developed countries and 27–90% of women in developing countries have been physically abused at least once [6]. Violence toward women is one of the issues and problems that have affected the lives of many women in the COVID-19 pandemic in various social classes and strata [9]. A study in France indicated that, 32% increase was reported in violence toward women in the first week of lockdown [10]. Since the gender is one of the important determining factors in violence toward women and mental health disorders.Women have a more significant psychological impact from the conditions of the pandemic [11]. On the other hand, addressing these issues is considered a global priority, but in Iran, no study has been done in this regard, this study was conducted to determine the relationship between mental health and violence toward women during the COVID-19 pandemic in Iran.

## Methods

### Design and ethical consideration

The present study is a descriptive correlational study. The ethics committee of Kerman University of Medical Sciences approved the study with the project No. 99,000,232 and the code of ethics No. IR.KMU.REC.1399.305. Participation in this study was voluntary. The study goals and procedures were explained to all participants, and the women’s informed consent was obtained.

### Sample and setting

This study was conducted on women in Kerman (southeast of Iran) during 2 months (Late-October to November 2020). Inclusion criteria were the ability to read and write in Persian and access to social media and the internet. Non-response to more than one third of the questionnaire was the exclusion criteria. Cochran’s formula with infinite population was used to calculate the sample size. Z = 1.96, *p* = q = 0.5, and d = 0.03 were considered. Therefore, 384 women were estimated for the present study. By taking into account a ten-percent dropout, 423 women were estimated.

### Measures

The researcher uploaded the electronic form of the questionnaires in WhatsApp groups. The questionnaires will be automatically sent to the correspond author’s e-mail after completion. Demographic and background information questionnaire, general health questionnaire (GHQ-12) and violence toward women inventory (VTWI) were used in this research.

General health questionnaire(GHQ-12) : The general health questionnaire (GHQ) is a self-administered screening questionnaire, designed for use in consulting settings aimed at detecting individuals with a diagnosable psychiatric disorder [12]. GHQ has been widely translated and used as a screening tool in many different languages, such as French [13], Chinese [14], English [15] and Persian [16]. A number of studies have reported psychometric characteristics of the GHQ-12 with Cronbach’s alpha coefficient values ranging from 0.75 to 0.9 in the unidimensional model. However, many studies have shown that GHQ-12 measures psychological morbidity in more than one dimension, most common being in two or three dimensions [15]. This scale consists of two sub-scales to measure mental disturbances and social performance disturbances. A 4-point likert-type scale (from 0 to 3) was used. The positive items were corrected from 0 (always) to 3 (never) and the negative ones from 3 (always) to 0 (never). The scores were used to generate overall score rating from 0 to 36, with higher scores indicating higher distress.

Violence toward women inventory (VTWI) examined the violence experienced by women during the past 12 months, includes 32 items and 4 subscales: psychological violence [1–16], physical violence [17–27], sexual violence [28–30] and economic violence [31, 32]. The inventory is rated on a 3-point likert scale (never/don’t know = 1, once = 2, twice or more = 3). The total score is between 32 and 96. The total scores for the subscales of psychological, physical, sexual and economic violence are 16–48, 11–33, 3–9 and 2–6, respectively. Cronbach’s alpha of this questionnaire is 0.97 [17, 18]. In Iran, Cronbach’s alpha coefficient for psychological abuse, physical abuse, sexual abuse, and economic abuse were estimated 0.90, 0.93, 0.79, and 0.78 respectively, and an alpha of 0.95 was found for the total questionnaire. The intra-cluster correlation index was 0.98 [19].

### Data analysis

According to Kolmogorov-Smirnov test, the data follows a normal distribution(*P* > 0.05), so descriptive (frequency, percent, mean and standard deviation) and inferential statistics (independent t- test, one-way analysis of variance and pearson correlation) with SPSS version 25 were used. Significance level was considered 0.05.

## Result

Findings of the study showed that the mean age of women was 33.9 ± 7.4, 48.3% of women had two children, 49.3% had bachelor’s degree and 54.8% were Employed. More than half of the participants had a monthly family income of 3–6 million.

The results of the independent t- test showed that there is no significant relationship between women’s mental health and job, also, the results of one-way analysis of variance showed that there is a significant relationship between women’s mental health and the number of children, family income and women’s age.

The results of independent t-test showed that violence toward women (VTW) is significant relationship with women’s job, so that violence was higher among employed women. Also, the results of one-way analysis of variance showed that VTW was significantly relationship with the number of children, women ‘s education, family income and age, so that VTW was higher in women with 3 children, high school degree, family income under 3 million and women over 40 years old (Table 1).

**Table 1:** Demographic characteristics of study sample and their associations with Mental Health and violence toward women (VTW)

**Group** **Variable**		Frequency (%)	Mental health		Violence toward women (VTW)	
			Mean (SD)	***P***	Mean (SD)	***P***
Job	Employed	219(54.8)	27.5(9.4)	t = -0.09 *P* = 0.92	56.3(13)	t = 6.22 *P* < 0.001
	Housewife	181(45.2)	27.6(9.8)		49(0.9)	
Number of children	1	113(28.2)	27.8(9.6)	F = 0.79 *P* = 0.49	54.7(14)	F = 3.2 *P* = 0.02
	2	193(48.3)	27.6(9.6)		52(8.8)	
	3	81(20.3)	26.6(9.7)		55.6(9.2)	
	More than 3	13(3.3)	30.7(8.9)		50(1)	
Education	High school	60(15)	25.2(8.5)	F = 1.47 *P* = 0.22	60.2(16.9)	F = 14.42 *P* < 0.001
	Diploma	67(16.8)	28.5(10)		52(4.2)	
	Bachelor	197(49.3)	27.9(9.7)		51.6(9.2)	
	More undergraduate	60(15)	27.7(7)		52.42(4.5)	
Family income per month	Under 3 million	93(23.3)	26.4(9.5)	F = 0.89 *P* = 0.41	55.50(12.2)	F = 9.9 *P* < 0.001
	3–6 million	221(55.3)	27.7(9.5)		50.65(4.3)	
	Above 6 million	86(21.5)	28.3(10)		51.12(9.7)	
Age	< 25	17(4.3)	23.7(9.9)	F = 0.820 *P* = 0.51	53(1)	F = 10.77 *P* < 0.001
	26–30	77(19.3)	27.7(9.7)		52.12(3.9)	
	31–35	153(38.3)	27.6(9.7)		52(12.6)	
	36–40	126(31.5)	27.5(9.1)		53.3(7.8)	
	> 40	27(6.8)	29(10.8)		65.6(16.7)	

Findings showed that the mean mental health of women at the COVID-19 pandemic was 15.14 ± 8.8. based on the results of the validity and reliability of the general health questionnaire (GHQ-12) conducted by Ebadi et al., the mental health cut-off point was determined to be 14.5 [33]. Women have moderate mental health during the COVID-19 pandemic. Findings of the study showed that the mean of VTW during the COVID-19 pandemic was 54.43 ± 10.6. The mean of psychological violence, physical violence, sexual violence and economic violence was respectively, 28.7 ± 8.9, 20.6 ± 7.7, 4.3 ± 1.8 and 2.7 ± 1.2 (Table don’t show). Also 88 participants (22%) had “Much more than usual” response to questions included :” Been able to face up to your problems?” and “ Been feeling unhappy and depressed?” (Table 2).

**Table 2:** The participants’ responses to Mental health questionnaire

Items	Responses (*N*/%)			
1. Been able to concentrate on what you’re doing?	**Better than usual**	**Same as usual**	**Less than usual**	**Much less than usual**
	90(22.5)	185(46.3)	88(22)	37(9.3)
2. Lost much sleep over worry?	**Not at all**	**No more than usual**	**Rather more than usual**	**Much more than usual**
	90(22.5)	131(32.8)	123(30.8)	56(14)
3. Felt you were playing a useful part in things?	**Better than usual**	**Same as usual**	**Less than usual**	**Much less than usual**
	74(18.5)	187(46.8)	67(16.8)	72(18)
4. Felt capable of making decisions about things?	**Better than usual**	**Same as usual**	**Less than usual**	**Much less than usual**
	72(18)	187(46.8)	69(17.3)	72(18)
5. Felt constantly under strain?	**Not at all**	**No more than usual**	**Rather more than usual**	**Much more than usual**
	66(16.5)	188(47)	68(17)	78(19.5)
6. Felt you couldn’t overcome your difficulties?	**Not at all**	**No more than usual**	**Rather more than usual**	**Much more than usual**
	66(16.5)	185(46.3)	77(19.3)	72(18)
7. Been able to enjoy your normal day-to-day activities?	**Better than usual**	**Same as usual**	**Less than usual**	**Much less than usual**
	67(16.8)	177(44.3)	79(19.8)	77(19.3)
8. Been able to face up to your problems?	**Better than usual**	**Same as usual**	**Less than usual**	**Much less than usual**
	56(14)	178(45)	78(19.5)	88(22)
9. Been feeling unhappy and depressed?	**Not at all**	**No more than usual**	**Rather more than usual**	**Much more than usual**
	55(13.8)	178(44.5)	79(19.8)	88(22)
10. Been losing confidence in yourself?	**Not at all**	**No more than usual**	**Rather more than usual**	**Much more than usual**
	61(15.3)	178(44.5)	78(19.5)	83(20.8)
11. Been thinking of yourself as a worthless person?	**Not at all**	**No more than usual**	**Rather more than usual**	**Much more than usual**
	56(14)	177(44.3)	81(20.3)	86(21.5)
12. Been feeling reasonably happy, all things considered	**Better than usual**	**Same as usual**	**Less than usual**	**Much less than usual**
	62(15.5)	182(45.5)	73(18.3)	83(20.8)

It is shown in the Table 3, in subscales of psychological violence % 28.2 of women had twice or more the experience of “Yelled at you during a heated argument?” and, %30 had the experience “Belittled your approach toward child rearing or accused you of being a failure as a wife and mother? ”.

In subscales of physical violence, %21.8 twice or more experience “Thrown, kicked, or broken something while arguing with you?” (Table 3).

**Table 3:** The participants’ responses to VTW ( subscales of psychological, physical violence)

	**Items**	**Responses (** ***N*** **/%)**		
		never/don’t know	once	twice or more
Psychological violence	1. Finished an argument and made his own decision about a matter that concerns both of you?	209(52.3)	111(27.8)	80(20.0)
	2. Yelled at you during a heated argument?	**106(26.5)**	**181(45.3)**	**113(28.2)**
	3. Insulted you, cursed you, used abusive language, or called you names?	**239(59.8)**	**84(21.0)**	**77(19.3)**
	4.Tried to prevent you from doing what you want (e.g., visiting relatives or friends)?	**283(70.8)**	**40(10.0)**	**77(19.3)**
	5. Given you dirty looks in an attempt to intimidate you?	**209(52.3)**	**108(27.0)**	**83(20.8)**
	6. Stormed out of the house after an argument, cursing and yelling at you?	**227(56.8)**	**110(27.5)**	**63(15.8)**
	7.Tried to control your behavior by investigating, interrogating, and following you?	**333(83.3)**	**30(7.5)**	**37(9.3)**
	8. Threatened to throw something at you and said things to intimidate you?	**310(77.5)**	**10(2.5)**	**80(20.0)**
	9. Accused you of paying more attention to and doing more for others than for him?	**220(55.0)**	**113(28.2)**	**67(16.8)**
	10. Degraded and insulted you or your acquaintances in an attempt to intimidate you?	**199(49.8)**	**104(26.0)**	**97(24.3)**
	11. Accused you of being lazy, indifferent, and failing to fulfill your obligations toward him and the household?	**264(66.0)**	**36(9.0)**	**100(25.0)**
	12. Requested or forced you to do something with the intention of insulting or humiliating you?	**330(82.5)**	**20(5.0)**	**50(12.5)**
	13. Belittled your approach toward child rearing or accused you of being a failure as a wife and mother?	**224(56.0)**	**56(14.0)**	**120(30.0)**
	14. Degraded your family, relatives, or friends by humiliating or cursing them?	257(64.3)	**47(11.8)**	**96(24.0)**
	15. Reprimanded or scolded you while belittling your thoughts, beliefs, and attitudes?	**263(65.8)**	**40(10.0)**	**97(24.3)**
Physical violence	16. Belittled the way you dress, your body, and the way you keep up your appearance?	**261(65.3)**	**89(22.3)**	**50(12.5)**
	17. Thrown, kicked, or broken something while arguing with you?	**256(64.0)**	**57(14.2)**	**87(21.8)**
	18. Pushed you, kicked you, or tried to knock you over?	**310(77.5)**	**23(5.8)**	**67(16.8)**
	19. Pushed or pulled you hard?	**320(80.0)**	**23(5.8)**	**57(14.2)**
	20. Threatened you with a knife or another sharp implement?	**373(93.3)**	**17(4.3)**	**10(2.5)**
	21. Slapped you?	**330(82.5)**	**33(8.3)**	**37(9.3)**
	22. Attacked you with his hands on different parts of your body?	**282(70.5)**	**47(11.8)**	**71(17.8)**
	23. Attacked you with a stick, a belt, or another object of that kind?	**353(88.3)**	**10(2.5)**	**37(9.3)**
	24. Tried to choke you or placed his arms around your neck in an attempt to harm you?	**373(93.3)**	**17(4.3)**	**10(2.5)**
	25. Pulled your hair or yanked your clothes?	**333(83.3)**	**20(5.0)**	**47(11.8)**
	26. Attacked you with household equipment (e.g., a chair)?	**363(90.8)**	**10(2.5)**	**27(6.8)**

Pearson correlation showed that there is no significant relationship between women’s mental health and VTW score. While findings showed that there is a weak inverse and significant relationship between women’s mental health and subscale of psychological and economic violence, so that with increasing psychological and economic violence, their mental health decreases. There was also no significant relationship between physical and sexual violence and women’s mental health (Table 4).

**Table 4:** The correlation between scores of VTW and Mental health

Variable	Mental Health Score	
	**Pearson correlation coefficient**	***P*** **-value**
Psychological Violence	-0.09	0.04
Physical Violence	-0.00	0.9
Sexual Violence	-0.40	0.42
Economic Violence	-0.09	0.04
Violence toward women (VTW)	-0.32	0.52

## Discussion

The aim of this study was to investigate the relationship between mental health and violence toward women(VTW)during the COVID-19 pandemic. The results showed that VTW was significantly relationship with job, so that violence was higher among employed women. This finding is consistent with the study of Fallah et al. (2016) [20]. Being employed by women can expose them to the disorder of the family center and the conflict of roles [21], on the other hand, employed women are less obedient to their husbands due to their financial independence [22], this can be the reason for the increase in the incidence of violence between couples, although cultural issues have an impact in this field [21]. Further investigation of this issue and accurate identification of factors is necessary.

In the present study, VTW was significantly relationship with the number of children, women’s education, family income and age, so that VTW was higher in women with 3 children, high school education and family income under 3 million and over 40 years old. Explaining this finding, it can be said that the higher the level of literacy and knowledge of women, it is considered as a protective factor against VTW because using skills such as anger control and problem solving skills greater their ability to deal with injuries and also awareness of social and family duties [20]. This finding is similar with the studies conducted in Iran [23] and Ethiopia [24].

Facing violence in women increases with the number of children. This can be due to the effect of economic well-being. When the husband cannot earn as much as others, they resort to violence in order not to lose their money.A study by Sezer Kisa in 2021 found that low family income increases the risk of VTW [25]. A study in Iran also showed that families with lower economic classes are more likely to use violence due to various economic pressures [26]. The results showed that VTW was higher in women over 40 years old, which is in line with the findings of Amini et al. (2014) [21]. This age period in women coincides with the retirement period of men, this period is usually a critical period with stress and anxiety due to changes in the socio-economic status of men and adaptation to new conditions and it can cause more conflicts in the family. Of course, more research is recommended in this area.

The findings of the present study showed that the higher a women’s education, the less violence toward her, which is consistent with the results of the study conducted by Hosseini et al. In 2019 [27]. Educated people, in their social learning, learn positive and constructive behavior with others, which does not affect the behavior of others towards them. Women with low social status are more likely to be exposed to violence [28]. Kisa et al. (2021) noted that low family income and education levels are two factors contributing to the increase in VTW [25].

The findings of the present study also showed that the mean mental health of women at the time of the COVID-19 pandemic was 15.14 ± 8.8. Due to the fact that the cut-off point for mental health is 14.5, women have moderate mental health during the COVID-19 pandemic. The majority of participants had “No more than usual” response to questions included :” Been able to face up to your problems?” and “ Been feeling unhappy and depressed?”.

A study conducted in China at the beginning of the COVID-19 pandemic in 2020 also showed that women were significantly more mental health disorders the COVID-19 pandemic [29]. In pandemic catastrophes around the world, acute mental disorders characterized by disturbing memories are more common in women than men. There is some evidence that fluctuations in hormone levels are responsible for changing sensitivity to emotional stimuli, which may be the basis and form the basis of specific vulnerability to mental disorder in women [30].

The findings also showed that the mean of VTW during the COVID-19 pandemic was 54.43 ± 10.6, indicating that VTW was moderate. On the other hand, among the dimensions of VTW during COVID-19 pandemic, the mean dimension of psychological violence is higher among other dimensions. A study by Almeida et al. (2020) also showed that psychological violence is higher among women during COVID-19 pandemic, which reduces mental health [9].

Pearson correlation showed that there is a significant inverse relationship between women’s mental health and subscale of psychological and economic violence, so that with the increase of psychological and economic violence, their mental health decreases. Hosseini et al.‘s study in Iran showed that during the COVID-19 pandemic and the closure of businesses and economic pressure on the family, of course, economic violence also follows, which in turn increases the psychological pressure on women and their mental health. In fact, it should be noted that men’s unemployment has a devastating effect on their behavioral status, which results in harm to the family environment and, in particular VTW [31].

According to studies, the COVID-19 pandemic and lockdown increase the risk of conflict between couples and violence, especially VTW [32]. Staying at home because of outbreak the COVID-19 pandemic undoubtedly creates power dynamics and can lead to VTW and make the home a place of insecurity.

Staying at home can provide more space to fight for power. In other words, staying at home due to the epidemic and the harmful conditions of this disease, causes the couple to stay together more and this increases the interactions between them; Now, when couples have power dynamics and also experience the stress of the disease, they may form destructive interactions and create violent relationships [34].

On the other hand, when family members experience stressful events outside the family, this stress can be drawn into the relationship and affect the quality of interactions [33]. The experience of stress is not specific to the couple and can be transmitted to other family members such as children, in which the stress of each member is transferred to another member, resulting in a cycle of dysfunctional interactions [35]. A brief look at the lives of abused women it indicates that their children have severe behavioral problems and abnormalities such as homelessness, aggression, depression, indifference to others and abnormal behaviors such as theft, addiction, etc. and affect their personal and social life in the future [36].

## Conclusion

In general, it can be concluded that during the COVID-19 pandemic, the level of Violence toward women was higher, which is an important threat to disrupt the mental health of women. Therefore, it is suggested that policy makers and planners, apart from the physical effects of the COVID-19 pandemic, pay attention to its psychological dimension, especially for women, and try to allocate funds to maintain and promote mental health and family.

Therefore, considering the role of awareness and attitude of people towards spousal abuse and violence toward women, it is suggested that the level of awareness and knowledge of married women and men about the destructive effects of violence toward women should be improved by various courses and workshops.

## Data Availability

The data that support the findings of this study are available from the ethics committee of the Kerman University of Medical Sciences but restrictions apply to the availability of these data, which were used under license for the current study, and so are not publicly available. Data are however available from the authors upon reasonable request and with permission of the ethics committee of the Kerman University of Medical Sciences.

## Abbreviations

GHQ-12: General Health Questionnaire
VTWI: Violence toward women inventory
